# The effectiveness of low‐level laser therapy as an adjunct to non‐surgical periodontal treatment: a meta‐analysis

**DOI:** 10.1111/jre.12361

**Published:** 2016-03-02

**Authors:** C. Ren, C. McGrath, L. Jin, C. Zhang, Y. Yang

**Affiliations:** ^1^ Faculty of Dentistrythe University of Hong KongHong Kong SARChina

**Keywords:** chronic periodontitis, low‐level laser therapy, non‐surgical periodontal treatment, systematic review

## Abstract

**Background and Objectives:**

Although low‐level laser therapy (LLLT) has been demonstrated to have a biomodulatory effect on periodontal tissue, no systematic review has exclusively addressed its effectiveness as an adjunct to non‐surgical periodontal treatment. This study aimed to evaluate whether an additional benefit exists for the application of LLLT compared with scaling and root planing (SRP) alone.

**Material and Methods:**

An extensive search was conducted in the Cochrane Library (Issue 8, 2015), PubMed (1997) and EMBASE (1947) before August 2015 for randomized controlled trials (RCTs). The bias risk was assessed with the Cochrane tool for risk of bias evaluation. A meta‐analysis was performed using REVMAN 5.3.

**Results:**

After independent screening of 354 initial records, eight publications (seven RCTs) were included. However, six were rated as ‘having a high risk of bias’ as a result of major methodological weakness in ‘allocation concealment’ and ‘blinding of key personnel’. Meta‐analysis showed that LLLT‐mediated SRP demonstrated significant short‐term benefits over SRP monotherapy in the improvement of the probing pocket depth (*p* = 0.0009 at 1 mo; *p* = 0.03 at 2 mo) and the level of interleukin‐1β in the gingival crevicular fluid (*p* = 0.01 at 1 mo). Nevertheless, LLLT failed to show significant additional intermediate‐term (3 and 6 mo) effects in terms of clinical parameters and alveolar bone density.

**Conclusion:**

These findings indicated that LLLT showed only short‐term additional benefits after conventional SRP. Its long‐term effects remain unclear due to substantial methodological weaknesses and an insufficient number of current studies. Future RCTs with better designs and longer follow‐up periods are required to assess the effectiveness of LLLT as an adjunctive treatment strategy in patients with periodontal disease.

For decades, periodontal disease has been a major challenge for oral health and quality of life 1. Chronic periodontitis is an inflammatory disease caused by infection with periodontopathic bacteria that results in the progressive destruction of the tooth‐supporting tissues and eventually tooth loss 2. It is recognized that non‐surgical periodontal treatment by subgingival scaling and root planing (SRP) remains the most effective approach to eliminating the source of infection 3. However, as an invasive approach, conventional mechanical SRP creates a wound in the already inflamed periodontal tissue, and the restoration of this tissue depends largely on favourable cellular and molecular responses 4, 5.

To strengthen the effects of non‐surgical periodontal treatment, high‐intensity laser irradiation using an Er:YAG laser, Nd:YAG laser or diode laser has been introduced for its potential benefits in the ablation of calculi and debridement of pockets and for its bactericidal effects 6, 7, 8. Nevertheless, its additional advantages have been challenged by the findings of several evidence‐based studies 9, 10. In contrast to the thermal effects of high‐power lasers, low‐level laser therapy (LLLT) is recommended for its photochemical role in anti‐inflammation, biostimulation and analgesia within the domains of low‐power output (within the mW range), low‐energy dosage (10^−2^–10^2^ J/cm^2^) and appropriate wavelengths (600–1000 nm) 11, 12, 13, 14. Whereas thermal lasers may cause damage to the root surface during cutting and ablation 15, almost no adverse events have been reported with the use of the low‐energy laser, also known as the soft or therapeutic laser, which is targeted mainly at soft tissue, and does not cause perceptible temperature changes 12, 13, 14. The most commonly used types of low‐level laser include the He‐Ne laser and the increasingly popular diode lasers (GaAlAs laser, InGaAlP laser, etc.) 12. Since its introduction in 1960s, LLLT has been widely used in various dental disciplines: postsurgical care, bone remodelling, neural restoration, orofacial pain relief and, more recently, the treatment of periodontal disease 12, 13, 14.

According to the Arndt–Schultz law, the desirable biological reactions must be triggered within a therapeutic window 16. Doses below that range are not sufficient to make a difference, and doses over that range may have inhibitory effects. Lasers with wavelengths in the red and near‐infrared range exhibit less absorption by water and tissue chromophores (haemoglobin and melanin), thus penetrate deeper into tissue (5–10 mm) 13, 16, 17. These properties make LLLT a promising treatment strategy for soft tissue wounds 11. It is believed that LLLT functions via the mitochondrial respiratory chain, resulting in the increased production of adenosine triphosphate and subsequently facilitating the proliferation of fibroblasts, release of growth factors and synthesis of collagen 12, 13, 18, 19. Meanwhile, *in vitro* and animal studies have shown that LLLT suppresses inflammation in periodontal tissue by modulation of the local immune response and by reducing the production and release of certain proinflammatory cytokines, such as tumour necrosis factor alpha (TNF‐α), interleukin‐1β (IL‐1β) and prostaglandin E_2_ 20, 21, 22, 23. In addition, LLLT has been found to improve the local microcirculation by angiogenesis and vasodilation, thus alleviating tissue oedema and inflammation 24.

However, there are differences in the results of the clinical trials that have investigated the additional benefits of LLLT in non‐surgical periodontal treatment 25, 26, 27, 28, 29, 30, 31, 32. Qadri *et al*. 31 found that adjunctive treatment with LLLT attenuated periodontal inflammation over the short term as assessed by the gingival index (GI), plaque index (PI), probing pocket depth (PPD) and matrix metalloproteinase‐8 level in the gingival crevicular fluid. However, Lai *et al*. 29 reported no significant improvement in any of their clinical parameters, namely, PPD, clinical attachment level or bleeding on probing (BOP), between LLLT‐mediated SRP and SRP monotherapy. Because no previous systematic review has exclusively addressed the effectiveness of LLLT as an adjunct to non‐surgical periodontal treatment, it is essential to conduct an evidence‐based study by comprehensive assessment of the accumulated data.

The objective of this systematic review was to evaluate on the basis of the results of randomized controlled trials (RCTs) whether LLLT, in combination with conventional mechanical debridement, provides any additional benefits over SRP alone as assessed by the clinical parameters (PPD as the primary outcome) and biochemical markers of periodontal inflammation (secondary outcomes).

## Material and methods

This systematic review was carried out in accordance with the Cochrane Handbook for Systematic Review of Interventions and the Preferred Reporting Items for Systematic Reviews and Meta‐Analysis (PRISMA) 33, 34.

### Search strategy

An extensive literature search was performed before August 2015 in the Cochrane Library (Issue 8, 2015), PubMed (1997) and EMBASE (1947). Any record relevant to RCTs of the adjunctive effects of LLLT in non‐surgical periodontal treatment was included for further screening with no restrictions regarding the publication year or language. The reference lists of all selected full‐text publications were scanned at the same time. No additional manual search of journals was performed. The search terms included ‘periodontitis’, ‘chronic periodontitis’, ‘periodontal disease’, ‘periodontal inflammation’, ‘gingival inflammation’, ‘periodontal treatment’, ‘dental scaling’, ‘scaling and root planing’ and ‘non‐surgical periodontal treatment’ for the diseases and circumstances under investigation; ‘laser irradiation’, ‘laser therapy’, ‘phototherapy’, ‘diode laser’, ‘biostimulation’, ‘low‐level laser’, ‘low‐intensity laser’, ‘low‐power laser’, ‘low‐energy laser’, ‘therapeutic laser’ and ‘soft laser’ for synonyms of LLLT, combined with outcomes of interest, including ‘plaque index’, ‘gingival index’, ‘probing pocket depth’, ‘clinical attachment level’, ‘bleeding on probing’, ‘gingival crevicular fluid’ and ‘biochemical markers’.

### Study selection

In the first stage, the titles and abstracts of all retrieved reports were screened for potentially eligible studies. The full‐text articles of the previously identified studies were then examined in detail according to predefined eligibility criteria for inclusion in the qualitative review. Finally, the references covered by the selected studies were searched manually to avoid the omission of any information related to the topic. Two reviewers performed the screening process independently. Whenever there was a disagreement between the two reviewers regarding study selection, discussions were carried out until a consensus was reached. The inter‐reviewer reliability was assessed by Cohen's kappa test, assuming 0.6 as an acceptable threshold value.

### Inclusion criteria

1. Studies included were RCTs that examined the adjunctive effects of LLLT in non‐surgical periodontal treatment.
2. Participants were patients with the diagnosis chronic periodontitis. There were no restrictions in the age, gender, ethnicity or socio‐economic status of the participants.
3. The participants were randomly allocated to the intervention group or to the control/placebo group. Each participant underwent conventional SRP (with an ultrasonic scaler and/or hand instrumentation) as their initial periodontal treatment. The intervention group underwent LLLT to the periodontal tissue of the target teeth after SRP. A sham laser was applied in the placebo group, and no laser was used in the control group.
4. The outcome variables included clinical indices of periodontal inflammation (i.e., PPD, clinical attachment level, PI, GI, etc.) and levels of biochemical or immunological markers (i.e., TNF‐α, IL‐1β and prostaglandin E_2_) in the gingival crevicular fluid or the periodontal tissue.

### Exclusion criteria

1. Potential participants who had any systematic disease or who were under medication that was known to affect the inflammation progress and wound healing of periodontal tissue were excluded, as were any who had undergone periodontal treatment within the past 6 mo.
2. Any studies in which high‐power thermal lasers (output power of 1 W or greater) were applied for calculus ablation, pocket debridement or bacteria reduction were excluded.
3. Any studies including photodynamic therapy involving a low‐level laser in conjunction with a photo‐sensitizer were excluded.

### Assessment of risk of bias

The risk of bias in the included studies was evaluated with reference to the Cochrane Tool for risk of bias assessment 33. This tool consists of seven evaluation domains, including ‘random sequence generation’, ‘allocation concealment’, ‘blinding of participants’, ‘blinding of key personnel’, ‘incomplete outcome data’, ‘selective reporting’ and ‘other bias’. The comprehensive methodological quality of each study was judged as low risk if all seven domains were rated as ‘having a low risk of bias’, as moderate risk if at least one domain was rated as ‘having an unclear risk of bias’ and as high risk if one or more domains was assessed as ‘having a high risk of bias’.

### Data extraction

The following data were extracted from the included studies: publication information, country, study design, sample size, subject characteristics (such as demographic characteristics, inclusion criteria for chronic periodontitis and smoking habits), randomization method, allocation concealment, blinding measures, intervention and placebo or control approach, laser parameters and regimen, outcome measurements, follow‐up duration, patients lost to follow‐up and the occurrence of any adverse events.

### Statistical analysis

The meta‐analysis was conducted using revman 5.3 33. The weight of each individual study included in the meta‐analysis for every effect estimate was determined by its reported standard deviation and sample size 33. The effect size was estimated and reported as the mean difference (MD) or standardized mean difference (SMD) with the 95% confidence interval (CI) for clinical indices and biochemical markers. Because each analysis had a small number of studies, the between‐studies variance was poorly estimated. Thus, a ‘fixed‐effect model’ was adopted for all analyses 34. Heterogeneity was assessed with a chi‐squared test and the *I* ^2^ statistic at an alpha level of 0.10. Moderate to substantial heterogeneity was considered to exist if the *I* ^2^ statistic was greater than 50%. The statistical significance level for the hypothesis test was set at an α level of 0.05 for two‐tailed *z* tests.

## Results

### Search and selection results

**Figure 1:**
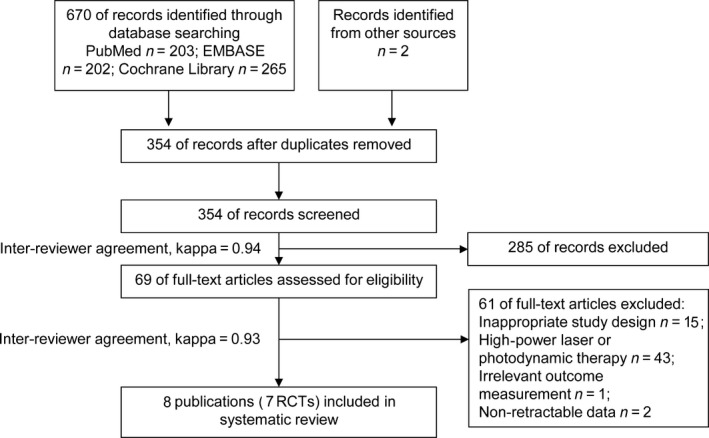
PRISMA flow diagram of the study inclusion process.

The process of study selection is shown in Fig. 1. At the beginning, 672 records were identified from the electronic and manual search. After the removal of duplicates, 354 publications remained for independent screening, of which 69 were deemed potentially eligible on the basis of their title and abstract (inter‐reviewer agreement, κ = 0.94). An additional 61 studies were excluded after scanning the full text (inter‐reviewer agreement, κ = 0.93). Thus, the entire procedure resulted in the inclusion of eight publications (seven RCTs) involving 180 participants from seven countries in the qualitative review 25, 26, 27, 28, 29, 30, 31, 32.

### Characteristics of included studies

The study characteristics and laser parameters are displayed in Tables 1 and 2, respectively. Three of the seven RCTs included in this study adopted a parallel‐arm design 25, 26, 27, 28, and the rest used a split‐mouth design 29, 30, 31, 32. There was great variation in the number of participants enrolled in each study (10–60). Despite a wide age range (22–70 years old), all of the studies but one 32 recruited adult patients. All of the participants were in good general health at the beginning of the study. Although all participants were diagnosed with chronic periodontitis, different criteria were used at recruitment. Six of the studies included patients with moderate to advanced chronic periodontitis 25, 26, 27, 28, 29, 30, 31, whereas one study recruited patients with mild chronic periodontitis 32. Most of the studies applied PPD as an inclusion criterion, with thresholds of 4–6 mm 28, 30, ≤ 7 mm 31, 4–10 mm 27 and ≥ 5 mm 29. Some studies also took into consideration tooth mobility 27, 31, site location 29 and angular bone defect 29 during sample selection and matching. Smokers were excluded by all but two studies 27, 31; one 27 included subjects who smoked more than 10 cigarettes per day and the other 31 gave no definition for smoking. Only one study 27 assessed the influence of smoking status on the effects of LLLT on the patients’ clinical parameters. Despite its less favourable effect on the change in the sulcus bleeding index, it was found that smoking status did not influence the effects of LLLT in the reduction of the PPD or clinical attachment level at a significant level. Meanwhile, adjunctive LLLT in smokers showed a positive intermediate‐term effect in the reduction of the sulcus bleeding index, the PPD and the clinical attachment level after SRP.

**Table 1:** Characteristics of included studies

Study ID	No. of subjects (M/F), target teeth and sites (anterior/posterior)	Age in mean ± SD (range) and Country	Inclusion criteria for CP	No. of smokers (M/F)	Study design	Treatment	Outcome measures	Evaluation interval and follow‐up
Angelov *et al*. 2009 & Pesevska *et al*. 2012 25, 26	I: 60 CP subjects (32/28) *I*_a_: 20, *I*_b_: 20, *I*_C_: 20; C: 20 periodontally healthy subjects	I: 48.9 (35–70) C: NR USA	Moderate or severe CP	Smokers not included	RCT (parallel)	I_a_: SRP I_b_: SRP+LLLT for 5 consecutive days I_c_: SRP+LLLT for 10 consecutive days C: no intervention	Clinical parameters: PI, GI, SBI biochemical markers: TNF‐α level in gingival tissue	Baseline and after treatment (10 d)
Aykol *et al*. 2011 27	36 CP subjects (22/14) I: 18 (7/11) C: 18 (7/11) 866 teeth I: 418, C: 448	I: 43.56 ± 6.70 (31–58) C:42.22 ± 7.53 (31–53) Turkey	1. moderate to advanced CP 2. PPD: 4–10 mm 3. Tooth mobility ≤ Grade III	18 smokers criteria: ≥10 cigarettes per day I: 9, C: 9	RCT (parallel)	I: SRP+LLLT C: SRP	Clinical parameters: PI, SBI, PPD, CAL biochemical markers: MMP‐1, TIMP‐1, TGF‐β1, and b‐FGF level in GCF	Baseline, 1, 3 and 6 mo after treatment (6 mo)
Calderín *et al*. 2013 28	27 CP subjects (12/15) I_a_: 9, I_b_: 9 C: 9	*I*_a:_ 52.89 ± 11.98 *I*_b_:50.44 ± 10.51 C:50.44 ± 15.91 Spain	1. moderate to advanced CP 2. PPD: 4–6 mm	Smokers not included	RCT (parallel)	I_a_: SRP+ single LLLT I_b_: SRP+ repetitive LLLT C: SRP	Clinical parameters: FMPS, FMBS, PPD, CAL biochemical markers: IL‐1β,TNF‐α, RANKL and OPG in GCF	Baseline, 4 and 8 wk after treatment (2 mo)
Lai *et al*. 2009 29	14 CP subjects (1/13) 55 teeth I: 28 C:27	43.6 (33–57) Hong Kong	1. moderate to advanced CP 2. at least two matched sites, one site from anterior region and the other from premolars 3. matched with PPD over 5 mm and comparable angular bone defects	Smokers not included	RCT (split‐mouth)	I: SRP+LLLT C: SRP	Clinical parameters: PL, PPD, PAL, Rec, BOP alveolar bone change: periapical radiographs (DSR) biochemical markers: GCF volume	Clinical parameters: baseline, 3, 6, 9, and 12 mo after treatment Biochemical markers and radiographs: baseline, 1, 3, 6, 9, and 12 mo after treatment (12 mo)
Makhlouf *et al*. 2012 30	16 CP subjects (4/12) 2–3 teeth per quadrant	(22–50) Egypt	1. CP 2. contralateral pockets with PPD of 4–6 mm in at least three teeth in each quadrant	Smokers not included	RCT (split‐mouth)	I: SRP + LLLT P: SRP+ sham laser	Clinical parameters: GI, PPD biochemical markers: IL‐1β in GCF bone density: intraoral radiographs	Clinical parameters and biochemical markers: baseline, 5 wk, 3 and 6 mo bone density: baseline, 6 and 12 mo (12 mo)
Qadri *et al*. 2005 31	17 CP subjects (7/10) I: 17 P: 17 170 teeth (13–17, 23–27 for each subject) I: 85 P: 85	53 (35–70) Sweden	1. moderate CP 2. PPD ≤7 mm 3. Tooth mobility ≤ Grade II	5 smokers	RCT (split‐mouth)	I: SRP+ LLLT P: SRP+ sham laser	Clinical parameters: PPD, GI, PI biochemical markers: GCF volume, elastase activity, IL‐1β, MMP‐8 level in GCF microbial analysis: subgingival plaque for 12 bacteria	Clinical parameters: baseline (before SRP), 1 wk after last LLLT; biochemical and microbial sampling: baseline (1 wk after SRP), 1 wk after last LLLT (2 mo)
Ribeiro *et al*. 2008 32	10 CP subjects 80 teeth (4 teeth per quadrant on the maxillary or mandibular arch) 480 sites (6 sites per tooth)	(15–35) Brazil	1. CP 2. PPD ≤ 4 mm	Smokers not included	RCT (split‐mouth)	I: SRP+ LLLT P: SRP+ sham laser	Clinical parameters: PPD, CAL, GI, pain level: VAS	Clinical parameters: baseline and 48 h after treatment, VAS: immediately after SRP (2 d)

b‐FGF, basic‐fibroblast growth factor levels, IL‐1β, interleukin‐1β; BOP, bleeding on probing; C, control group; CAL, clinical attachment level; CP, chronic periodontitis; DSR, digital subtraction radiography; F, female; FMBS, full‐mouth bleeding score; FMPS, full‐mouth plaque score; GCF, gingival crevicular fluid; GI, gingival index; GR, gingival recession; I, intervention group; IL‐1α, interleukin‐1α; M, male; MMP‐1, matrix metalloproteinase‐1; MMP‐8, matrix metalloproteinase‐8; MMP‐9, matrix metalloproteinase‐9; OPG, osteoprotegerin; P, placebo group; PAL, probing attachment level; PBI, papillary bleeding index; PI, plaque index; PL, supragingival plaque; PPD, probing pocket depth; RANKL, receptor activator of nuclear factor κΒ ligand; Rec, change in recession; SBI, sulcus bleeding index; TGF‐β1, transforming growth factor‐β1; TIMP‐1, tissue inhibitor matrix metalloproteinase‐1; TNF‐α, tumour necrosis factor‐α; VAS, visual analogue scale.

**Table 2:** The parameter and regimen of diode laser applied in included studies

Study ID	Type of laser	Wavelength	Beam size	Output/energy (density)	Dosage per irradiation or tooth	Time of exposure	Method of application	Frequency of laser treatment	Accumulative dosage (dose × frequency)
Angelov, *et al*. 2009 & Pesevska, *et al*. 2012 25, 26	Diode laser, continuous mode	630–670 nm	0.2 cm^2^	25 mW, 1.875 J/cm^2^	0.375 J/irradiation 0.75 J/tooth	15 s/irradiation 4 min/quadrant	Applied externally with a light contact to the gingival tissue corresponding to the treated pockets, scanning in apical‐coronal direction in all interdental spaces on both facial and lingual surfaces	5 and 10 consecutive days after SRP (5 or 10 sessions)	1.875 or 3.75 J/irradiation; 3.75 or 7.5 J/tooth
Aykol, *et al*. 2011 27	GaAlAs laser, continuous mode	808 nm	0.28 cm^2^	250 mW, 4 J/cm^2^	1–2 J/irradiation 1–2 J/tooth	Incisors and premolars: 10 s/irradiation Molar: 20 s/irradiation	Non‐contact with a distance of 0.5 to 1 cm	Day 1, 2, 7 after SRP (3 sessions)	3–6 J/irradiation; 3–6 J tooth
Calderín, *et al*. 2013 28	Diode laser, continuous mode	670 nm	NR	200 mW	6 J/irradiation 12 J/tooth	30 s/irradiation 60 s/tooth	Inserted into the pocket no deeper than the probing depth, and gently moved all along the sulcus of buccal, lingual and interproximal surfaces	Single LLLT: Day 1 after SRP; Repeated LLLT: Day 1, 2, 4, 7, and 11 after SRP (1 or 5 sessions)	6 or 30 J/irradiation; 12 or 60 J/tooth
Lai, *et al*. 2009 29	He‐Ne laser, continuous mode	632 nm	0.07 cm^2^	0.2 mW, 2.83 mW/cm^2^, 1.7 J/cm^2^	0.12 J/irradiation 0.12 J/tooth	600 s/irradiation 600 s/tooth	The optical fiber was placed perpendicularly to the interdental papilla and kept in place touching the buccal gingival surfaces.	8 sessions over the following 3 mo (8 sessions)	0.96 J/irradiation; 0.96 J/tooth
Makhlouf, *et al*. 2012 30	GaAlAs laser, continuous mode	830 nm	0.03 cm^2^ Irradiated area: 1 cm^2^	100 mW, 3 W/cm^2^, 3 J/cm^2^	3 J/irradiation 6 J/tooth	30 s/irradiation 60 s/tooth	The tip was positioned externally at the base of the pocket lingually and buccally in slight contact, perpendicularly to the long axis of the tooth, in a sweeping motion.	3 sessions during the 1st and 2nd wk, followed by 2 sessions during the 3rd wk, and then once weekly during the 4th and 5th wk (10 sessions)	30 J/irradiation; 60 J/tooth
Qadri, *et al*. 2005 31	Diode laser	635 nm 830 nm	0.2 cm^2^	635 nm: 10 mW, 50 mW/cm^2^, 4.5 J/cm^2^; 830 nm: 70 mW, 350 mW/cm^2^, 8.75 J/cm^2^	Papillae: 0.9 J/irradiation apically: 1.75 J/irradiation Total: 5.3 J/tooth	635 nm: 90 s/irradiation 830 nm: 25 s/irradiation 230 s/tooth	Gingival papillae were treated with 635 nm laser and 6 mm more apically with 830 nm laser from the buccal and lingual sides with slight contact.	Once a week for 6 wk after SRP (6 sessions)	5.4 or 10.5 J/irradiation; 31.8 J/tooth
Ribeiro, *et al*. 2008 32	GaAlAs laser	780 nm (analgesia) 660 nm (wound healing)	0.04 cm^2^	Pre‐ and post‐ surgery analgesia: 70 mW, 35 J/cm^2^; wound healing: 35 mW, 8.8 J/cm^2^	Analgesia: 1.4 J/irradiation 2.8 J/tooth Would healing: 0.35 J/irradiation 0.35 J/tooth	Analgesia: 20 s/irradiation (tooth) Wound healing: 10 s/irradiation (tooth)	Laser with 780 nm wavelength was punctually applied to the papillae and cervical region, to an apical point for analgesia. Laser with 660 nm wavelength was applied to the papillae and cervical region for drainage and tissue repair	Before and immediately after SRP, 24 and 48 h later (3 sessions)	Analgesia: 4.2 J/irradiation; 8.4 J/tooth; Wound healing: 1.05 J/irradiation; 1.05J/tooth

Mechanical debridement, including supragingival and/or subgingival SRP combined with instruction on oral hygiene was performed for all participants as their initial periodontal treatment in a single 27, 30 or multiple sessions 25, 26, 28, 29. A diode laser with a wavelength ranging from 630 to 830 nm was used in most studies. Nevertheless, the output power and exposure time of LLLT diverged greatly among studies, leading to an energy dosage ranging from 0.12 to 12 J per tooth. Irradiation was applied externally in slight contact with the buccal and lingual gingival surface in a static or scanning manner, whereas in one study 28 the irradiation penetrated into the pocket via a diffusing tip. Although all of the studies included multiple sessions of irradiation, a large variation in regimens was observed. Irradiation frequencies varied from 4 to 10 sessions within 3 mo after SRP, which yielded an accumulative dosage of 1–30 J per site (1–60 J per tooth). Interestingly, one study compared the effects of a single session with multiple sessions and obtained an outcome favouring the latter in terms of the reduction in inflammatory mediators 28. A pseudo laser was used in three studies to ensure the blinding of the participants 30, 31, 32.

Each of the RCTs examined clinical parameters, and the levels of biochemical markers in the gingival crevicular fluid were also assessed in four studies 27, 28, 30, 31. One study assessed the level of TNF‐α in gingival biopsies 25, 26. The alveolar bone density was measured by radiography in two studies 29, 30. Microbial analysis of the subgingival plaque was performed in one study 31; no significant difference was seen in the percentage of positive samples between the laser and placebo groups. In addition, one study compared the pain levels immediately after treatment and found no significant difference between the intervention and placebo groups 32. Notably, only three studies included more than 6 mo of follow‐up 27, 29, 30, and the others merely recorded short‐term outcomes 25, 26, 28, 31, 32. No adverse events were reported during follow‐up in any of the included studies.

### Assessment of methodological quality

**Figure 2:**
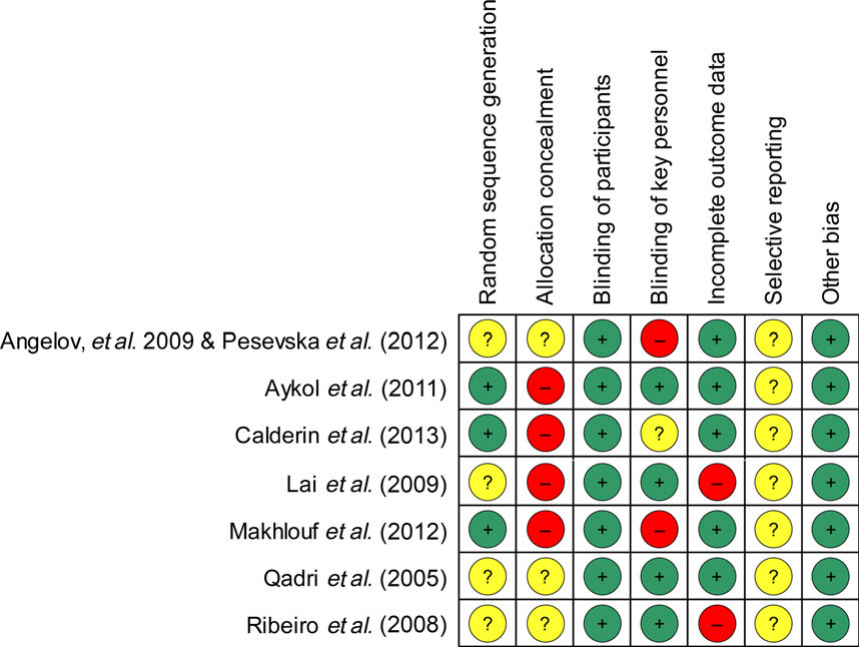
Risk of bias summary: review authors’ judgments about each risk of bias item for each included study.

**Figure 3:**
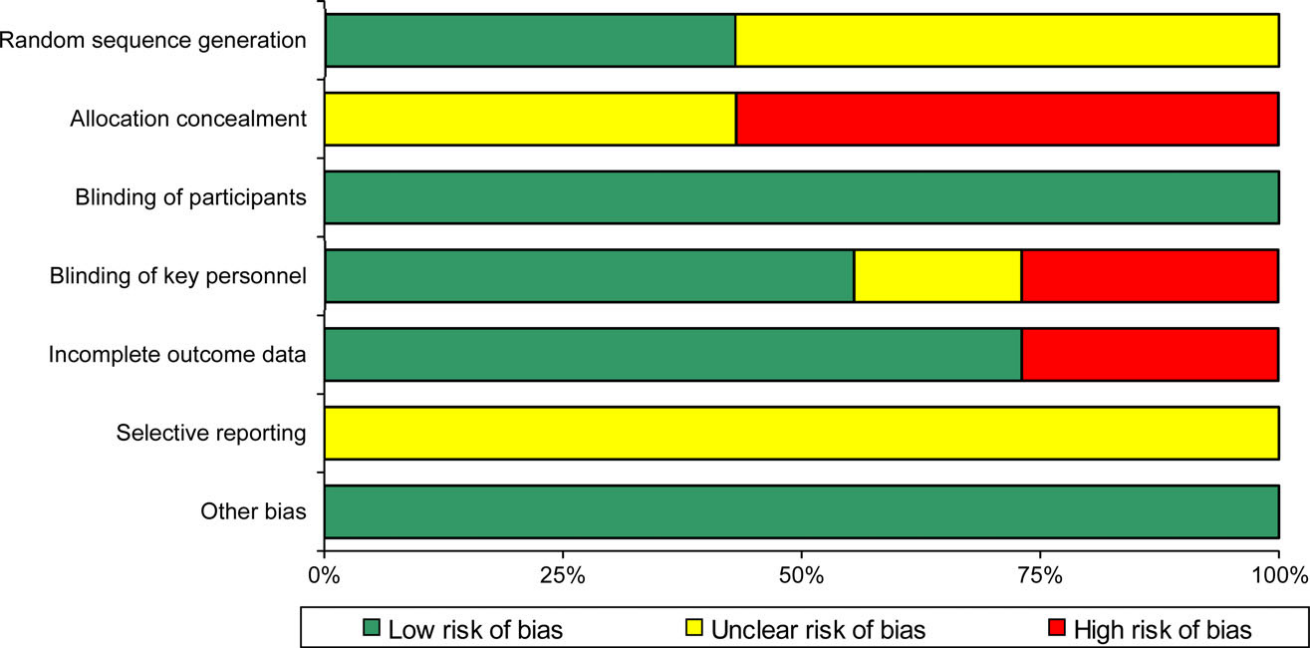
Risk of bias graph: review authors’ judgments about each risk of bias item presented as percentages across all included studies.

As shown in Fig. 2, only one study 31 was assessed as having a moderate risk of bias with six studies 25, 26, 27, 28, 29, 30, 32 as having a high risk of bias. When analysed according to different domains, the methodological weakness of the pooled evidence was mainly attributed to ‘allocation concealment’ and ‘blinding of key personnel’ (Fig. 3). Although all of the studies were presented as RCTs, only three described the manner in which the random sequence was generated 27, 28, 30. Four of the included RCTs failed to implement strict allocation concealment to prevent foreknowledge of the random sequence 27, 28, 29, 30, and the rest did not mention this issue explicitly 25, 26, 31, 32. Because clinical indices and biochemical markers were the objective outcome measures, the effect of a lack of true blinding of participants on the study results was considered insignificant. However, the reliability of the results may be at risk if key investigators who recruited patients, performed SRP or assessed the outcome data were aware of the grouping information. It was found that effective masking of key personnel was neglected in two studies 25, 26, 30 and was not described clearly in another one 28. Some patients were lost to follow‐up in two studies 29, 32 without appropriate explanation or management. There was insufficient information to assess whether the outcomes were reported selectively in any of the included studies.

### Effect of intervention

#### Short‐term effects

Four studies 27, 28, 30, 31 provided adequate data on clinical parameters including the PI, PPD and clinical attachment level, along with the amount of IL‐1β in the gingival crevicular fluid, which was obtained 1 or 2 mo after treatment. A meta‐analysis was thus conducted to assess the short‐term adjunctive effects of LLLT (Table 3). The PPD was significantly lower in the LLLT‐mediated group than in the SRP group at 1 mo (MD, −0.40; 95% CI, −0.64 to −0.17; *p* = 0.0009) and at 2 mo (MD, −0.28; 95% CI, −0.54 to −0.03; *p* = 0.03). Meanwhile, in comparison with the control group, a marginal improvement in the PI was observed in the LLLT‐adjunctive group (MD, −0.22; 95% CI, −0.44 to 0; *p* = 0.06). With regard to inflammatory cytokines, LLLT produced a significant additional effect in the reduction of IL‐1β levels in the gingival crevicular fluid at 1 mo (SMD, −0.77; 95% CI, −1.35 to −0.18; *p* = 0.01). However, no significant difference was detected in favour of the adjunctive use of LLLT with regard to the clinical attachment level (MD, −0.21; 95% CI, −1.08 to 0.67; *p* = 0.65).

**Table 3:** Meta‐analysis of LLLT's short‐term additional effects, comparison: SRP+LLLT versus SRP, outcome: clinical parameters (PI, PPD, CAL) and biochemical markers (IL‐1β) at 1 and 2 mo

Evaluation interval	Outcome	Studies	Number of participants	Model	Test for total effect			Test for heterogeneity	
					MD/SMD	95% CI	*p* Value	*I* ^2^ value (%)	*p* Value
1 mo	PI	27, 30	68	Fixed	−0.22	−0.44 to 0.00	0.06	0	0.32
	PPD	27, 28, 30	86	Fixed	−0.40	−0.64 to −0.17	0.0009*	0	0.65
	CAL	27, 28	54	Fixed	−0.21	−1.08 to 0.67	0.65	0	0.89
	IL‐1β	28, 30	50	Fixed	−0.77^a^	−1.35 to −0.18	0.01*	0	0.43
2 mo	PPD	28, 31	52	Fixed	−0.28	−0.54 to −0.03	0.03*	86	0.009

MD, mean difference; SMD, standardized mean difference; CI, confidence interval; PI, plaque index; PPD, probing pocket depth; CAL, clinical attachment level; IL‐1β, Interleukin‐1β.

a Intervention effect reported as SMD.

*p < 0.05, significant difference between SRP + LLLT and SRP.

#### Intermediate‐term effects

As shown in Table 4, intermediate‐term evaluations were made in three studies 27, 29, 30 at 3 and 6 mo. Compared with SRP alone, the use of LLLT as an adjunct provided no significant improvement in the PI (MD, −0.03; 95% CI, −0.32 to 0.26; *p* = 0.84 for 3 mo; MD, −0.08; 95% CI, −0.27 to 0.10; *p* = 0.39 for 6 mo), the PPD (MD, −0.28; 95% CI, −0.56 to 0.01; *p* = 0.06 for 3 mo; MD, −0.01; 95% CI, −0.15 to 0.12; *p* = 0.88 for 6 mo) or the clinical attachment level (MD, 0.07; 95% CI, −0.58 to 0.71; *p* = 0.84 for 3 mo; MD, 0.04; 95% CI, −0.62 to 0.69; *p* = 0.91 for 6 mo). No significant difference was seen in the alveolar bone density between the group with adjunctive LLLT and the SRP group (SMD, 0.18; 95% CI, −0.33 to 0.69; *p* = 0.48 for 6 mo).

**Table 4:** Meta‐analysis of LLLT's intermediate‐term additional effects, comparison: SRP+LLLT versus SRP, outcome: clinical parameters (PI, PPD, CAL) and alveolar bone density at 3 and 6 mo

Evaluation interval	Outcome	Studies	Number of participants	Model	Test for total effect			Test for heterogeneity	
					MD/SMD	95% CI	*P* value	*I* ^2^ value (%)	*P* value
3 mo	PI	27, 30	68	Fixed	−0.03	−0.32 to 0.26	0.84	0	0.69
	PPD	27, 29, 30	96	Fixed	−0.28	−0.56 to 0.01	0.06	0	0.83
	CAL	27, 29	64	Fixed	0.07	−0.58 to 0.71	0.84	0	0.53
6 mo	PI	27, 30	68	Fixed	−0.08	−0.27 to 0.10	0.39	0	0.69
	PPD	27, 29, 30	96	Fixed	−0.01	−0.15 to 0.12	0.88	0	0.74
	CAL	27, 29	64	Fixed	0.04	−0.62 to 0.69	0.91	0	0.88
	Alveolar bone density	29, 30	60	Fixed	0.18^a^	−0.33 to 0.69	0.48	0	0.40

MD, mean difference; SMD, standardized mean difference; CI, confidence interval; PI, plaque index; PPD, probing pocket depth; CAL, clinical attachment level.

a Intervention effect reported as SMD.

## Discussion

LLLT has long been recommended to facilitate wound healing because it is a non‐invasive therapy with biostimulatory and anti‐inflammatory properties 11, 12, 13, 14. However, previous systematic reviews have either focused on the application of thermal lasers in periodontal treatment or assessed the clinical effects of high‐power and low‐power lasers as a whole 9, 10, 35, 36. Few evidence‐based studies can be found to clarify whether LLLT adds benefit to the traditional non‐surgical periodontal treatment. Therefore, this systematic review was conducted to elucidate this research question. The current body of evidence indicates that LLLT in conjunction with SRP shows some short‐term superiority as assessed by the PPD, but it appears equivalent to SRP monotherapy in the intermediate term. However, the results of this systematic review should be interpreted with caution because of the considerable methodological shortcomings and substantial heterogeneity among the included studies. Several factors require much attention before research can be conducted and decisions made.

### Sample selection and matching of intervention arms

Great variation was noted in the inclusion criteria, which covered mild, moderate and advanced categories of chronic periodontitis (Table 1). Moreover, some studies made judgments on the basis of comparable pocket depth, and some supplemented this criterion with tooth mobility and bone level. Nevertheless, one study 25, 26 failed to describe clearly their diagnostic and inclusion criteria. The pocket depth has been shown as a critical indicator of the effectiveness of non‐surgical periodontal treatment 37. Greater reductions in the PPD and gains in the clinical attachment level are expected in patients with deeper pockets 29. However, only one study 27 analysed the effects of LLLT according to subgroups of patients with moderate (4–6 mm) and deep (6–10 mm) pockets. LLLT was found to be effective in the reduction of PPD in both subgroups in comparison with their counterparts in the control group. Meanwhile, it appeared equally beneficial for the reduction of the clinical attachment level between subgroups at 6 mo 27. However, this conclusion should be applied carefully because no further clues could be obtained to support the balanced distribution of the two levels of pocket depth between the experiment and control groups mentioned above. Smoking is considered another principal factor that has a negative effect on the prognosis of periodontal disease 38. All of the studies addressed this issue at recruitment. However, only one study gave an explicit definition of the smoking status and matched the smokers between the laser and control groups 27. The smokers and non‐smokers were analysed and compared as subgroups in this study. LLLT was found to produce additional favourable effects on the clinical parameters among the smoking subjects. This effect was attributed to the positive role of LLLT in the microcirculation, in the synthesis of collagen and in cytokine modulation, which are negatively affected by smoking 27. At the same time, LLLT was shown to produce comparable effects in the reduction of the PPD and the clinical attachment level between smokers and non‐smokers 27. Notably, none of the included studies conducted a calculation of sample size beforehand to estimate the minimum number of subjects needed to detect a significant difference between the groups. These defects in sample selection and group matching may put the reliability of research outcomes at risk.

### Intervention and control measures

Although SRP was implemented in all of the participants as the initial therapy, there was no clear description of the post‐debridement maintenance. Only one study conducted strict long‐term oral hygiene instruction to ensure that only participants with good oral hygiene status and compliance were enrolled 30. It is known that good oral hygiene control serves as a prerequisite for successful treatment outcomes 3. Thus, explicit predefined criteria should be set to make the results comparable between groups and studies.

It is believed that the efficacy of laser therapy depends on a combination of parameters, including the wavelength, spot size, output power, energy dosage, exposure time and irradiation frequency 39. The wavelength plays a key role in laser–tissue interaction by modulation of the scattering and absorption characteristics 17, 39. Meanwhile, a biphasic dose response is considered to influence the clinical effectiveness of LLLT, which indicates the presence of a therapeutic window for optimal tissue reaction 16. Despite the efforts of accumulating *in vivo* and *in vitro* studies, the exact dosage range remains controversial 19, 25, 26, 27, 28, 29, 30, 31, 32, 40, 41. Some researchers recommended an energy dose of 1–10 J/cm^2^ for periodontal tissue 40, 42, 43. Substantial heterogeneity was seen in the laser parameters and regimens among the included studies, with wavelengths ranging from 630 to 830 nm, output powers ranging from 0.2 to 250 mW, energy densities ranging from 1.7 to 24 J/cm^2^, and application frequencies ranging from 4 to 10 sessions within 3 mo after SRP (Table 2). However, given the insufficient number of studies included, no sensitivity analysis or meta‐regression could be conducted to explore the effects of the laser parameters on their clinical effects. Interestingly, one study 28 compared the effects of a single session of irradiation with multiple LLLTs and reached a conclusion in favour of the multiple application method in the reduction of proinflammatory mediators, which is in agreement with some current perspectives 12, 16, 44. It was indicated that regular irradiation during the periodontal treatment course may produce more favourable effects. Remarkably, important parameters such as the spot size, energy dosage and application method were not detailed in some studies 27, 28, which undermined the quality of the collective evidence.

### Study design and outcome assessment

It is believed that the host response plays a key role in the progression of periodontal disease 45. By this token, a split‐mouth design serves as a good choice by which to eliminate intersubject variance, which is difficult to control even with perfect matching. In addition, a smaller sample size is required under this circumstance to achieve equal test power 46. In consideration of these merits, over half of the included studies adopted a contralateral control model 29, 30, 31, 32. Nevertheless, it must be borne in mind that a carryover effect would diminish the true effects of intervention that are under investigation 47. Thus, the rationale of the split‐mouth design should be based on a lack of verified systematic effects of LLLT. Owing to the small number of studies included, no subgroup analysis could be performed to examine the differences in the effects of intervention between the studies with split‐mouth and parallel‐arm designs.

A remarkable shortcoming was seen in the methods of the included studies as assessed by the Cochrane Tool for risk of bias evaluation (Figs 2 and 3). Major drawbacks were observed to lie in ‘allocation concealment’ and ‘blinding of key personnel’. More than half of the studies included were conducted without effective measures to protect the allocation sequence 27, 28, 29, 30, and two failed to mask the outcome assessors or clinical operators 25, 26, 30. Furthermore, the methods of random sequence generation were not described explicitly in four of the included studies, which could have potentially biased the results 25, 26, 29, 31, 32.

Instead of calculus ablation and bacteria reduction, the effects of LLLT are mainly shown as photochemical and photobiological, which may play a larger role in the maintenance and healing processes of periodontitis 12. Thus, as a key indicator for the outcome of non‐surgical periodontal treatment, the PPD was chosen as the primary outcome and other clinical periodontal indices (clinical attachment level, PI and GI) along with gingival crevicular fluid levels of biochemical markers (IL‐1β) as the secondary outcomes in this review. In terms of clinical indices, only a short‐term trend was observed in favour of the LLLT‐adjunctive group in PPD reduction (Table 3). LLLT also showed some short‐term additional benefits in the reduction of plaque and improvement of the gingival condition 25, 26, 27, 31. However, with further examination of the intermediate‐term effects, no significant differences were revealed in the improvement of the clinical parameters or the alveolar bone density between LLLT‐mediated SRP and SRP monotherapy (Table 4). Thus, it was speculated that, after traditional SRP, the adjunctive effects of LLLT on the modulation of acute gingival inflammation and the alleviation of tissue oedema account for the extra decrease in pocket depth in the short term 12. This speculation was strengthened by the demonstrated effects of LLLT on the reduction of IL‐1β levels in the gingival crevicular fluid (Table 3). Multiple proinflammatory cytokines in the gingival crevicular fluid (i.e., IL‐1, IL‐6 and TNF‐α) have been found to correlate closely with the status of periodontitis; this finding greatly benefits the diagnosis, treatment and prognosis of periodontal disease 48. Although they have already been documented by a number of *in vitro* studies 20, 21, 22, 23, the effects of LLLT on the levels of proinflammatory cytokines in the gingival crevicular fluid appeared controversial among the clinical trials. For the first time, biochemical markers were assessed quantitatively in relevant systematic reviews. Considering the great variation in the selected cytokines and evaluation time‐points among the limited number of studies, only the data regarding the short‐term levels of IL‐1β in the gingival crevicular fluid could be synthesized and analysed. Thus, the exact effects of LLLT on the inflammatory mediators require further verification. More importantly, because it takes months or even years for the periodontal tissue to restore and maintain health after mechanical therapy, most studies adopted a follow‐up duration of less than 6 mo, leaving unexplored the long‐term effects of LLLT 49.

### Implications for future research

Given the weaknesses of the current evidence identified in this review, the following suggestions are proposed for future clinical studies. Initially, the inclusion criteria of periodontal patients should be carefully designed before the experiment and clearly reported in the manuscript. Factors that may influence disease progression, such as the pocket depth and smoking status, should be taken into account when recruiting and matching participants for both the split‐mouth and parallel‐arm designs. Sample‐size estimation is advisable for RCTs. Moreover, it is important that both intervention and control measures follow predefined guidelines to reduce bias. It is suggested that laser parameters be chosen based on existing evidence and reported in a standardized and detailed manner. In addition, the risks and benefits should be balanced and discussed regardless of which study design is chosen. In addition, effective measures should be taken to reduce the risk of bias in the study methods, with special attention paid to allocation concealment and blinding. Last but not least, longer durations of follow‐up, adequate irradiation regimens and further exploration of biochemical markers are anticipated.

## Conclusions

Although LLLT is widely recommended for its biostimulatory and anti‐inflammatory roles, it only showed additional short‐term merits in reducing the pocket depth after conventional SRP. However, its intermediate‐term effects were found to be non‐significant. Its long‐term adjunctive benefits remain unclear because of the substantial methodological weaknesses and the insufficient number of existing studies. Future RCTs with better study designs, adequate sample power and longer durations of follow‐up are required to assess the effectiveness of LLLT as an adjunctive treatment strategy in patients with periodontal disease.

## Conflict of interest statement

None declared.

## Supporting information


**Figure S1.** Comparison: SRP + LLLT versus SRP; Outcome: PI; Evaluation time‐point: 3 mo.
**Figure S2.** Comparison: SRP + LLLT versus SRP; Outcome: PPD; Evaluation time‐point: 3 mo; Subgroup analysis: end score and change of score from baseline.
**Figure S3.** Comparison: SRP + LLLT versus SRP; Outcome: CAL; Evaluation time‐point: 3 mo.
**Figure S4.** Comparison: SRP + LLLT versus SRP; Outcome: PI; Evaluation time‐point: 6 mo.
**Figure S5.** Comparison: SRP + LLLT versus SRP; Outcome: PPD; Evaluation time‐point: 6 mo; Subgroup analysis: end score and change of score from baseline.
**Figure S6.** Comparison: SRP + LLLT versus SRP; Outcome: CAL; Evaluation time‐point: 6 mo.
**Figure S7.** Comparison: SRP + LLLT versus SRP; Outcome: alveolar bone density; Evaluation time‐point: 6 mo.
