# Supplementary material for: The effectiveness of low‐level laser therapy as an adjunct to non‐surgical periodontal treatment: a meta‐analysis
Source: J Periodontal Res. 2016 Mar 2;52(1):8–20. doi: 10.1111/jre.12361 (PMC5297978; doi:10.1111/jre.12361)
Supplement: Supplementary file 1 — Figure S1. Comparison: SRP + LLLT versus SRP; Outcome: PI; Evaluation time‐point: 3 mo. Figure S2. Comparison: SRP + LLLT versus SRP; Outcome: PPD; Evaluation time‐point: 3 mo; Subgroup analysis: end score and change of score from baseline. Figure S3. Comparison: SRP + LLLT versus SRP; Outcome: CAL; Evaluation time‐point: 3 mo. Figure S4. Comparison: SRP + LLLT versus SRP; Outcome: PI; Evaluation time‐point: 6 mo. Figure S5. Comparison: SRP + LLLT versus SRP; Outcome: PPD; Evaluation time‐point: 6 mo; Subgroup analysis: end score and change of score from baseline. Figure S6. Comparison: SRP + LLLT versus SRP; Outcome: CAL; Evaluation time‐point: 6 mo. Figure S7. Comparison: SRP + LLLT versus SRP; Outcome: alveolar bone density; Evaluation time‐point: 6 mo. [file JRE-52-8-s001.pdf]

Supplementary Material

Evaluation of LLLT's intermediate-term effects shown by forest plots (3 and 6 months)

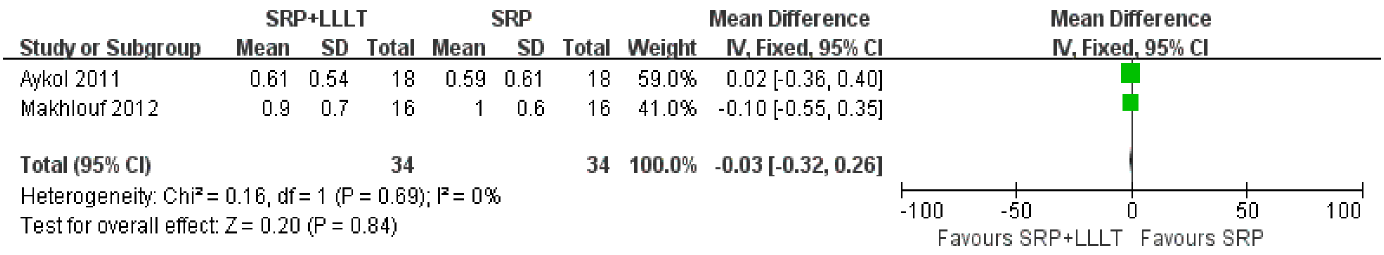

Fig. 1 Comparison: SRP+LLLT versus SRP; Outcome: PI; Evaluation time-point: 3 months

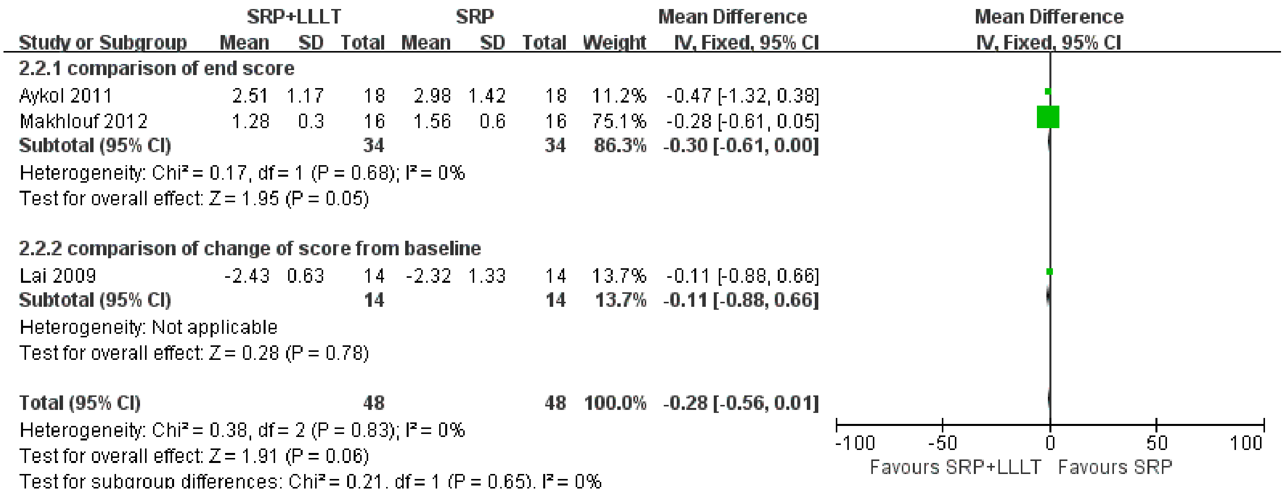

Fig. 2 Comparison: SRP+LLLT versus SRP; Outcome: PPD; Evaluation time-point: 3 months; Subgroup analysis: end score and change of score from baseline

Study or Subgroup	SRP+LLLT Mean	SRP+LLLT SD	SRP+LLLT Total	SRP Mean	SRP SD	SRP Total	Weight	Mean Difference IV, Fixed, 95% CI
Aykol 2011	1.1	1.09	18	0.9	1.26	18	70.6%	0.20 [-0.57, 0.97]
Lai 2009	2.04	1.57	14	2.29	1.65	14	29.4%	-0.25 [-1.44, 0.94]
**Total (95% CI)**			**32**			**32**	**100.0%**	**0.07 [-0.58, 0.71]**

Heterogeneity: Chi² = 0.39, df = 1 (P = 0.53); I² = 0%
Test for overall effect: Z = 0.21 (P = 0.84)

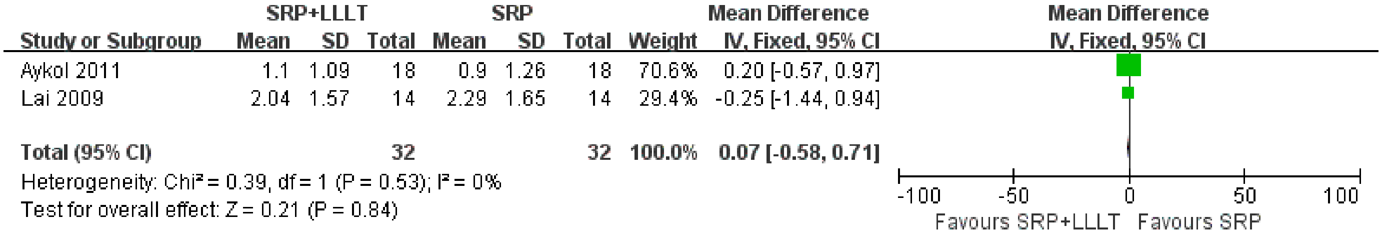

Fig. 3 Comparison: SRP+LLLT versus SRP; Outcome: CAL; Evaluation time-point: 3 months

Study or Subgroup	SRP+LLLT Mean	SRP+LLLT SD	SRP+LLLT Total	SRP Mean	SRP SD	SRP Total	Weight	Mean Difference IV, Fixed, 95% CI
Aykol 2011	0.67	0.6	18	0.68	0.62	18	21.4%	-0.01 [-0.41, 0.39]
Makhlouf 2012	0.48	0.3	16	0.58	0.3	16	78.6%	-0.10 [-0.31, 0.11]
**Total (95% CI)**			**34**			**34**	**100.0%**	**-0.08 [-0.27, 0.10]**

Heterogeneity: Chi² = 0.15, df = 1 (P = 0.69); I² = 0%
Test for overall effect: Z = 0.86 (P = 0.39)

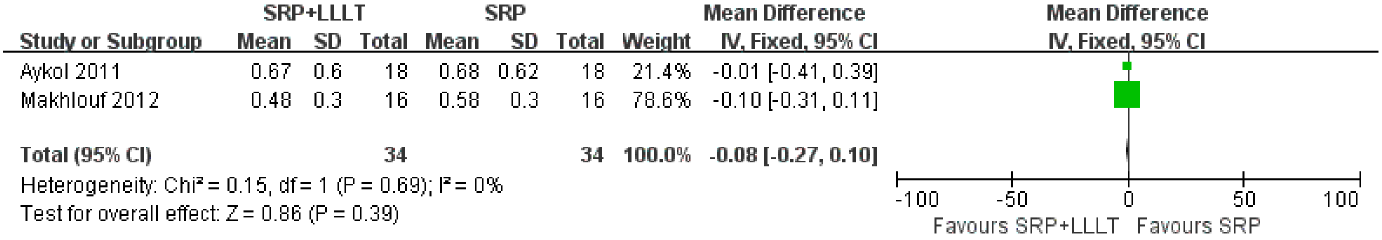

Fig. 4 Comparison: SRP+LLLT versus SRP; Outcome: PI; Evaluation time-point: 6 months

Study or Subgroup	SRP+LLLT Mean	SRP+LLLT SD	SRP+LLLT Total	SRP Mean	SRP SD	SRP Total	Weight	Mean Difference IV, Fixed, 95% CI
**3.2.1 comparison of end score**								
Aykol 2011	2.48	1.2	18	2.82	1.45	18	2.4%	-0.34 [-1.21, 0.53]
Makhlouf 2012	1.19	0.2	16	1.19	0.2	16	94.9%	0.00 [-0.14, 0.14]
**Subtotal (95% CI)**			**34**			**34**	**97.3%**	**-0.01 [-0.15, 0.13]**
Heterogeneity: Chi² = 0.57, df = 1 (P = 0.45); I² = 0%								
Test for overall effect: Z = 0.12 (P = 0.90)								
**3.2.2 comparison of change of score from baseline**								
Lai 2009	-2.46	1.04	14	-2.39	1.17	14	2.7%	-0.07 [-0.89, 0.75]
**Subtotal (95% CI)**			**14**			**14**	**2.7%**	**-0.07 [-0.89, 0.75]**
Heterogeneity: Not applicable								
Test for overall effect: Z = 0.17 (P = 0.87)								
**Total (95% CI)**			**48**			**48**	**100.0%**	**-0.01 [-0.15, 0.12]**

Heterogeneity: Chi² = 0.59, df = 2 (P = 0.74); I² = 0%
Test for overall effect: Z = 0.15 (P = 0.88)
Test for subgroup differences: Chi² = 0.02, df = 1 (P = 0.88), I² = 0%

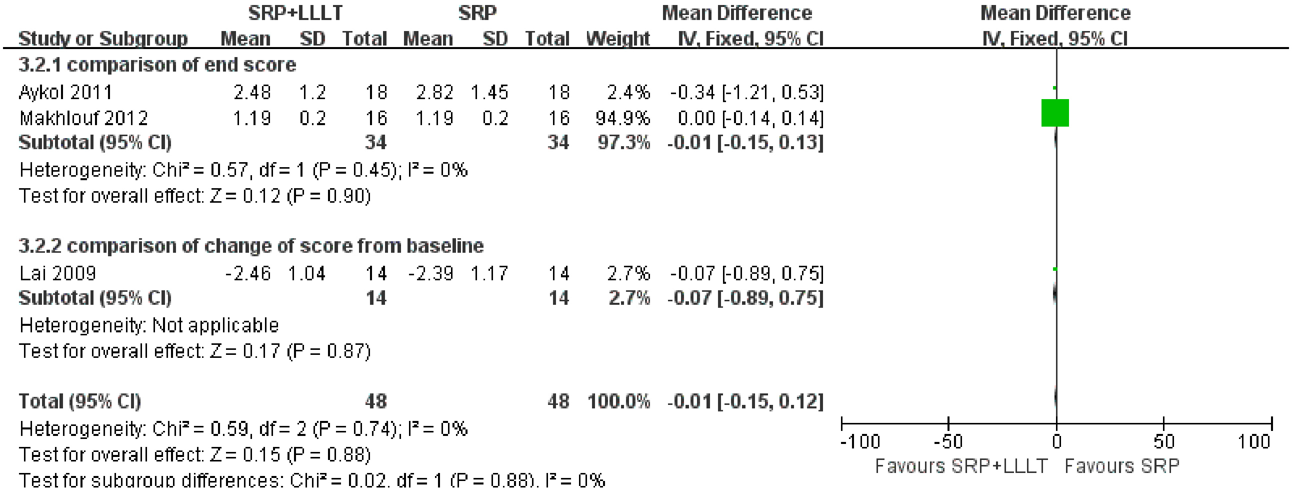

Fig. 5 Comparison: SRP+LLLT versus SRP; Outcome: PPD; Evaluation time-point: 6 months; Subgroup analysis: end score and change of score from baseline

	SRP+LLLT			SRP				Mean Difference	Mean Difference
Study or Subgroup	Mean	SD	Total	Mean	SD	Total	Weight	IV, Fixed, 95% CI	IV, Fixed, 95% CI
Aykol 2011	1.17	1.13	18	1.1	1.25	18	71.0%	0.07 [-0.71, 0.85]	
Lai 2009	2.21	1.55	14	2.25	1.73	14	29.0%	-0.04 [-1.26, 1.18]	
**Total (95% CI)**			**32**			**32**	**100.0%**	**0.04 [-0.62, 0.69]**	

Heterogeneity: Chi² = 0.02, df = 1 (P = 0.88); I² = 0%
Test for overall effect: Z = 0.11 (P = 0.91)

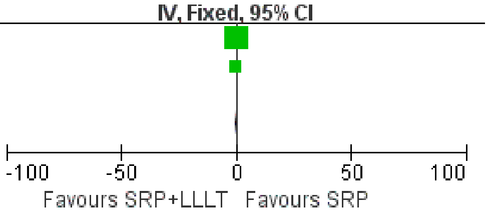

Fig. 6 Comparison: SRP+LLLT versus SRP; Outcome: CAL; Evaluation time-point: 6 months

	SRP+LLLT			SRP				Std. Mean Difference	Std. Mean Difference
Study or Subgroup	Mean	SD	Total	Mean	SD	Total	Weight	IV, Fixed, 95% CI	IV, Fixed, 95% CI
Lai 2009	309.29	261.88	14	322.44	240.09	14	47.2%	-0.05 [-0.79, 0.69]	
Makhlouf 2012	113.6	34.8	16	101.8	23.1	16	52.8%	0.39 [-0.31, 1.09]	
**Total (95% CI)**			**30**			**30**	**100.0%**	**0.18 [-0.33, 0.69]**	

Heterogeneity: Chi² = 0.72, df = 1 (P = 0.40); I² = 0%
Test for overall effect: Z = 0.70 (P = 0.48)

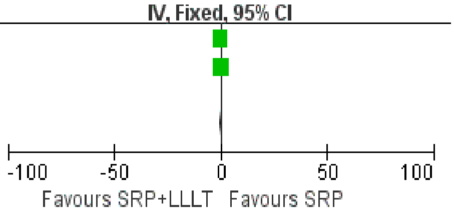

Fig. 7 Comparison: SRP+LLLT versus SRP; Outcome: alveolar bone density; Evaluation time-point: 6 months
